# The impact of ionic contribution to dielectric permittivity in 11CB liquid crystal and its colloids with BaTiO_3_ nanoparticles

**DOI:** 10.1140/epje/s10189-022-00228-9

**Published:** 2022-09-08

**Authors:** Joanna Łoś, Aleksandra Drozd-Rzoska, Sylwester J. Rzoska, Krzysztof Czupryński

**Affiliations:** 1 Institute of High Pressure Physics, Polish Academy of Sciences, X-PressMatter Lab, ul. Sokołowska 29/37, 01-142 Warsaw, Poland; 2 Faculty of Advanced Technologies and Chemistry, Military University of Technology, ul. gen. Sylwestra Kaliskiego 2, 00-908 Warsaw, Poland

## Abstract

The report shows the temperature behavior of the real part of dielectric permittivity in the static (dielectric constant) and low-frequency (LF) domains in bulk samples of 11CB and its BaTiO_3_-based nanocolloids. The study covers the isotropic liquid (I), nematic (N), smectic A (SmA), and solid crystal (Cr) phases. For each phase, the dominance of pretransitional fluctuations, significantly moderated by nanoparticles, is shown. The authors consider separate focuses on the dielectric constant $\varepsilon \left( {\varvec{T}} \right)$ evolution in the static domain, yielding mainly response from permanent dipole moment and its arrangement, and in the low-frequency (LF) domain $\Delta \varepsilon^{\prime } \left( f \right) = \varepsilon^{\prime } \left( f \right) - \varepsilon$ (where $\varepsilon^{\prime } \left( {\varvec{f}} \right)$ is for the real part of dielectric permittivity in the LF domain), which is associated solely with ionic-related polarization mechanisms. All of these led to new experimental evidence concerning I–N, N–SmA, and SmA–solid transitions, focusing on the strength and extent of pretransitional effects, critical exponents, and phase transitions discontinuities. The strong evidence for pretransitional effects near the SmA–Cr transition is notable, particularly regarding $\Delta \varepsilon^{\user2{^{\prime}}} \left( {{\varvec{f}},{\varvec{T}}} \right)$. Studies are supplemented by the discussion of DC electric conductivity—a parameter also related to the LF domain. Finally, the validity of the relation $\varepsilon^{\prime } \left( {\varvec{f}} \right) = {\varvec{Af}}^{ - 3/2} + \varepsilon$ (where *f* stands for frequency, and *A* is a constant parameter), often used for discussing dielectric spectra in LC compound and its nanocolloids in the LF domain, is examined.

**Graphical abstract:**

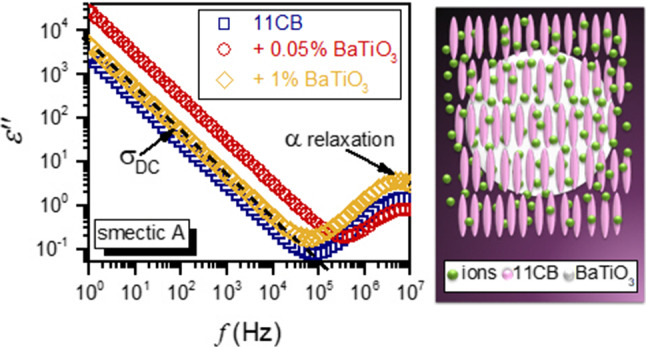

## Introduction

Liquid crystalline (LC) materials are essential for a myriad of applications due to their extraordinary sensitivity to external disturbances, especially the electric field [1–3]. LC compounds also constitute a unique experimental model system for fundamental research [1, 2, 4]. Regarding the latter, the isotropic liquid (I) phase of rod-like LC is considered a model for studying the vitrification and glass transition phenomena [4–10]. Notable are similarities between smectic A (SmA)–nematic (N) transition and those occurring in superconductors [11, 12]. The freezing of single symmetry elements in subsequent mesophases on cooling also draws the researchers' attention [1, 4].

Nanoparticles (NPs) are a unique type of solids for which the reduction in grain size below 100 nm leads to the appearance of new and unique properties—strongly manifested in interactions with surrounding matrix systems [13]. Liquid crystals are especially suitable for this role due to the mentioned extraordinary sensitivity even to a minor disturbance. The research carried out over several decades has shown unusual properties of LC-based nanocolloids (low concentrations of NPs) and nanocomposites (higher concentrations of NPs), leading to the emergence of a new research area in the field of liquid crystals and soft matter physics [14, 15]. Notwithstanding, the area of LC + NPs systems is still on the exploratory path beginning.

For LC or LC + NPs, the interaction with the external electric field is essential [1, 2], which indicates the primary role of broadband dielectric spectroscopy (BDS) [16] as the experimental monitoring method. Studies of the dielectric constant $\left( \varepsilon \right)$, directly related to the ability of molecules to interact with the electric field, are particularly important. Temperature evolution of the dielectric constant allows to discern the prevalence of different arrangements of permanent dipole moments coupled to molecules, namely: ${\text{d}}\varepsilon /{\text{d}}T < 0$ is related to the ‘parallel’ arrangement, i.e., dipole moments follow the direction of the electric field, and ${\text{d}}\varepsilon /{\text{d}}T > 0$ is for their antiparallel ordering [17]. Both in LC and LC + NPs systems, studies of dielectric constant and related properties revealed the dominant influence of multimolecular pretransitional fluctuations in the isotropic liquid and LC mesophases [5, 7, 18–21]. It is manifested via long-range pretransitional effects associated with the weakly discontinuous character of ${\text{Isotropic}} \leftrightarrow {\text{Nematic}}$, ${\text{Isotropic}} \leftrightarrow {\text{Smectic}}$, and ${\text{Nematic}} \leftrightarrow {\text{Smectic}}$ phase transitions. Such behavior is manifested via pretransitional effects explained within the *Physics of Critical Phenomena* [4]. For dielectric constant the following general relation describing such behavior can be concluded [18–24]:

1 $$ \varepsilon \left( T \right) = \varepsilon^{*} + a\left| {T - T^{*} } \right| + A\left| {T - T^{*} } \right|^{1 - \alpha } $$

where $\varepsilon^{*}$ and $T^{*}$ coordinates of the extrapolated continuous phase transition point located beyond the mesophase in which the given pretransitional effect is scanned; in the isotropic phase $T > T^{C} = T^{*} + \Delta T^{*}$, where $T^{C}$ is the clearing temperature, i.e., the isotropic–LC mesophase transition temperature, $\Delta T^{*}$ is the metric of the phase transition discontinuity, $a$ and $A$ are constant parameters, and the exponent $\alpha$ is related to the specific heat pretransitional anomaly.

Equation 1 was first introduced in refs. [18, 22], by the analogy to the behavior occurring in the homogeneous phase of critical binary mixtures of limited miscibility. This behavior was theoretically derived by Oxtoby et al. [25], using the droplet model approximation for precritical fluctuations, and by Sengers et al. [26], considering the internal energy of the near-critical liquids under the electric field. Only recently, the new approach offering the common description of pretransitional effects in the isotropic liquid phase on approaching the nematic phase in LC and the orientationally disordered crystals (ODIC) phase in plastic crystalline (PC) materials was proposed [27, 28]. The origins of Eq. 1 are also based on the reasoning by Mistura [29] and Fisher [30], leading to the conclusion that for critical fluids, the isochoric heat capacity $C_{V} \propto {\text{d}}\varepsilon /{\text{d}}T \propto \left( {T - T_{C} } \right)^{ - \alpha }$. The latter implemented to Eq. (1) yields:

2 $$ \frac{{{\text{d}}\varepsilon \left( T \right)}}{{{\text{d}}T}} = a + A\left( {1 - \alpha} \right)\left| {T - T^{*} } \right|^{ - \alpha } = a + A^{\prime } \left| {T - T^{*} } \right|^{ - \alpha } . $$

The coherent occurrence of Eqs. (1) and (2) were experimentally confirmed for N/SmA ← I [18, 22], SmA → I [23, 24], and recently ${\text{SmA}} - N$ transitions [31]. It is also notable that the derivative-based analysis (Eq. 2) enable the local distortions-sensitive insight into the evolution described by Eq. (1). It also reduces the number of fitting parameter. The coherent analysis of both $\varepsilon \left( T \right)$ and ${\text{d}}\varepsilon \left( T \right)/{\text{d}}T$ dependences facilitate reaching optimal values of fitted parameters [31]. Importantly, Eq. 2 contains fewer parameters than its hypothetical parallel for the heat capacity.

**Fig. 1:**
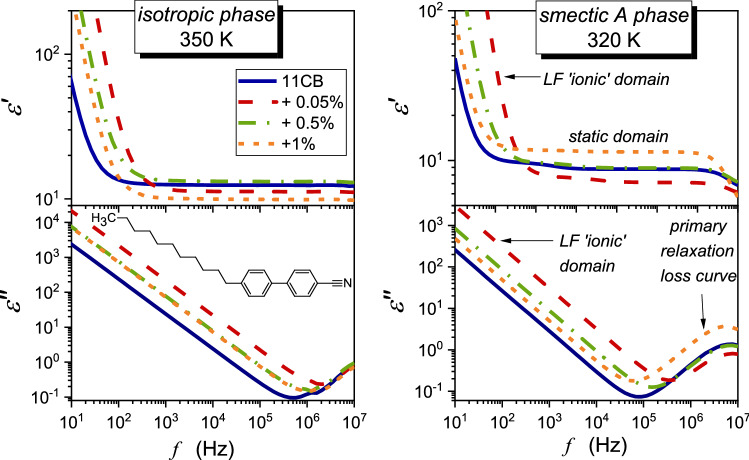
Examples of BDS spectra—the real and imaginary parts of dielectric permittivity as a function of frequency collected for 11CB and its BaTiO_3_-based nanocolloids. Characteristic features of the spectrum are marked, and 11CB molecular structure is shown

In wide-range temperature studies covering a few phases, selecting the proper reference frequency for determining the dielectric constant is essential. It requires broadband dielectric spectroscopy scans, presented as real and imaginary components of dielectric permittivity. Such results for undecylcyanobiphenyl (11CB) tested in the given report are shown in Fig. 1.

The horizontal part of $\varepsilon^{\prime } \left( f \right)$ is the static domain related to the dielectric constant, i.e., $\varepsilon^{\prime } \left( f \right) = \varepsilon = \varepsilon_{S} + \varepsilon_{\infty }$, where $\varepsilon_{S}$ and $\varepsilon_{\infty }$ are the dipolar and electron molecular contributions, respectively. In high-frequency range, $\varepsilon^{\prime } \left( f \right)$ strongly decreases since the component $\varepsilon_{S}$ diminishes [16, 17]. The crossover frequency domain is associated with the strong manifestation of the primary loss curve $\varepsilon^{\prime \prime } \left( f \right)$. Its peak is related to the primary relaxation time $\tau = 1/2\pi f_{{{\text{peak}}}}$ associated with the molecular reaction to the electric field. Peak height (maximum, $\varepsilon_{peak}^{\prime \prime }$) is the metric of coupled energy absorption [16, 31]. Loss curve branches describe the distribution of relaxation time. All these properties are influenced by pretransitional fluctuations, at least in the isotropic liquid phase. The cognitive gap for the impact of pretransitional fluctuations on dielectric properties in the low-frequency (LF) domain still exists. This region is dominated by transport processes associated with ionic species, both $\varepsilon^{\prime } \left( f \right)$, and $\varepsilon^{\prime \prime } \left( f \right)$ strongly increase on decreasing the frequency, as visible in Fig. 1. For discussing this behavior, two relations are explored in LC materials and their nanocomposites [32–40]:

3 $$ \varepsilon^{\prime } \left( f \right) = Af^{ - 3/2} + \varepsilon = \left( {\frac{{nq^{2} D^{3/2} }}{{\pi^{3/2}\varepsilon_{0} Lk_{B} T}}} \right)f^{ - 3/2} + \varepsilon $$

4 $$ \varepsilon^{\prime \prime } \left( f \right) = Bf^{ - 1} = \left( {\frac{{nq^{2} D}}{{\pi \varepsilon_{0} k_{B} T}}} \right)f^{ - 1} $$

where *n* is the mobile ions concentration, *q* denotes the charge of an ion, *D* is the constant related to diffusion, $\varepsilon_{0}$ is the free space permittivity, $k_{B}$ is the Boltzmann constant, *L* is the thickness of the dielectric cell (capacitor), and *T* is the absolute temperature.

The validity of Eq. 4 is obvious when recalling the basic definition of DC electric conductivity, namely: $\sigma = \omega \varepsilon^{\prime \prime } \left( f \right) = 2\pi f\varepsilon^{\prime \prime } \left( f \right)$ [16, 17]. Distortions from Eq. 3 can yield insight into possible low-frequency relaxation or the Maxwell–Wagner polarization processes [16]. The authors have not found any reliable experimental test or explanation validating Eq. 3, for the real part of dielectric permittivity in the LF domain.

To the best of the authors' knowledge, the only report focusing on the LF domain and the impact of pretransitional fluctuations considered the isotropic liquid phase of pentylcyanobiphenyl (5CB) [41]. It showed the following parameterizations for the temperature evolution [41]:

5 $$ \varepsilon^{\prime } \left( {T,f < f_{{{\text{static}}}} } \right) = \left[ {\varepsilon^{*} + a\left| {T - T^{*} } \right| + A\left| {T - T^{*} } \right|^{1 - \alpha } } \right]_{{{\text{static}}}} + \left[ {b\left| {T - T^{ \wedge } } \right|} \right]_{{{\text{ionic}}}} $$

6 $$ \Delta \varepsilon^{\prime } \left( {T,f} \right) = \varepsilon^{\prime } \left( {T,f < f_{{{\text{static}}}} } \right) - \varepsilon \left( T \right) = bT + bT^{ \wedge } = bT + B $$

where $b$ is the amplitude and $T^{ \wedge }$ is the frequency-dependent singular temperature.

In the static domain, the last term in Eq. 5 diminishes. Notable that the linear behavior described by Eq. 6 terminates ∼ 40 K above the clearing temperature, where fluctuations shrink to 2–3 molecules [20, 21, 41].

This report expands the use of the above-mentioned method to the case of smectic A and solid crystal phases. This method was adopted for pure LC compound (11CB) and nanocolloids. The results presented are supplemented with DC electric conductivity $\sigma \left( T \right)$ evolution insight, related to $\varepsilon^{\prime \prime } \left( f \right)$ behavior in the LF domain. Obtained BDS spectra enabled the examination of Eqs. (3) and (4).

## Experimental

Studies were carried out in 4-undecyl-4′-cyanobiphenyl (11CB) with ${\text{Crystal}} \leftrightarrow 326.15\,{\text{K}} \leftrightarrow SmA \leftrightarrow 330.16\,{\text{K}} \leftrightarrow {\text{Nematic}} \leftrightarrow 330.65\,{\text{K}} \leftrightarrow {\text{Isotropic}}$ mesomorphism [1]. The compound was synthesized and deeply cleaned by the LC team at the Military University of Technology, Warsaw, Poland. 11CB molecule is approximately rod-like, as shown in Fig. 1, and has a longitudinal permanent dipole moment: ***µ*** = 4.78 D [42]. Nonlinear dielectric effect (NDE) studies enable the reliable estimation of the phase transition temperature and discontinuity of the isotropic liquid to LC mesophase phase transition (Δ*T* = 5.6 K) via the linear regression fit to the temperature dependence of the reciprocal of NDE [43].

Paraelectric BaTiO_3_ nanopowder (diameter *d* = 50 nm) was purchased from US Research Nanomaterials, Inc. [44]. Mixtures of liquid crystal and nanoparticles were sonicated at a temperature higher than the isotropic to nematic phase transition for 4 h to obtain homogeneous suspensions. Concentrations of nanoparticles are given in weight fraction percentage (wt%).

Samples were placed in a flat-parallel capacitor (diameter *2r* = 20 mm) made of Invar with the distance between plates *d* = 0.15 mm. The voltage of the measuring field *U* = 1 V was applied. BDS studies were carried out using Novocontrol Alpha-A analyzer in the frequency range from 1 Hz to 10 MHz. Temperature was controlled using a Novocontrol Quatro cryosystem. Examples of dielectric permittivity spectra collected for the tested compound are shown in Fig. 1. It also indicates significant features of such spectra. As the reference frequency for determining the dielectric constant $f = 116\, {\text{kHz}}$ was chosen: $\varepsilon = \varepsilon^{\prime } \left( {f = 116\,{\text{kHz}}} \right)$.

Studies of rod-like LC compounds are often carried out under conditions yielding insight into two components of the dielectric constant—transverse ($\varepsilon_{ \bot }$) and longitudinal ($\varepsilon _{//}$) to the short and long molecular axis of the molecule. It is reached via the flat-parallel capacitor in which the tested sample is oriented by the (very) strong magnetic field. The alternative experimental path is a measurement in the capacitor with an adequately treated plate surface, supporting the required orientation of rod-like molecules, and with a tiny, micrometric gap between plates [1, 2, 4]. However, the orientation via an external magnetic field is possible only in the nematic phase. Thin-layer measurements, forcing the preferred direction, are possible in any phase, but the introduced constraints change generic features of the isotropic and smectic phases. Consequently, such studies are beyond the scope of the given report, which focuses on the LF properties of the ‘native’ phase, which is possible only using large (‘bulk’) capacitor gap and non-treated plates.

## Results and discussion

**Fig. 2:**
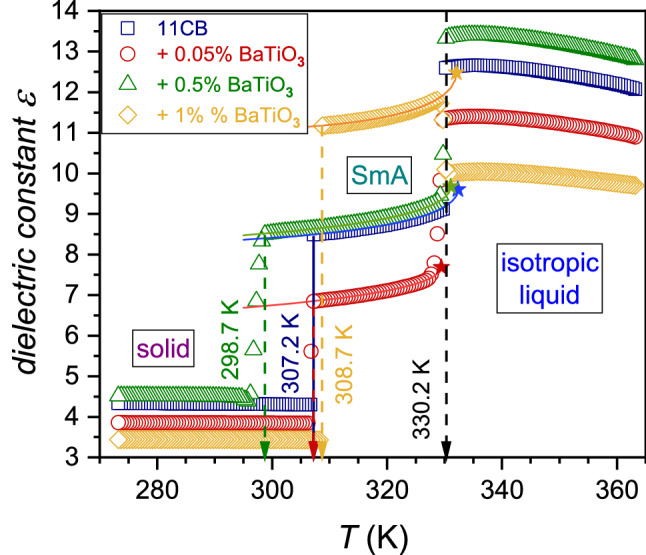
Temperature dependencies of dielectric constant in 11CB and 11CB + BaTiO_3_ nanocolloids. Arrows indicate characteristic temperatures $T^{C}$ and $T_{m}$. The real part of the dielectric permittivity measured at $f = 116\,{\text{kHz}}$ was chosen as the dielectric constant $\left( {\varepsilon^{\prime } \left( {T,f = 116\,{\text{kHz}}} \right) = \varepsilon } \right)$. Curves portraying data are related to Eq. (1), with parameters given in Table 2. Stars show the hypothetical continuous phase transition point

**Fig. 3:**
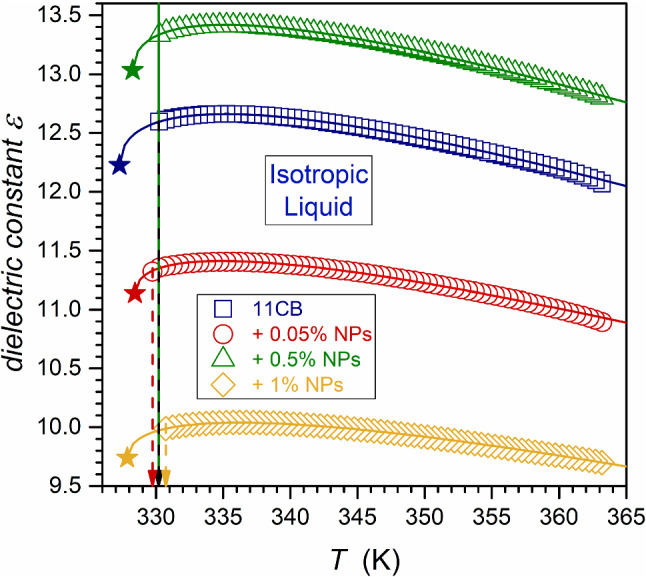
Temperature dependencies of dielectric constant in the isotropic liquid phase of 11CB and its nanocolloids with BaTiO_3_. Curves portraying data are related to Eq. (1), with parameters given in Table 1. Stars show the hypothetical continuous phase transition points. Arrows indicate clearing temperatures for isotropic liquid–LC mesophase transitions

Figure 2 shows the temperature dependences of dielectric constant in 11CB and its nanocolloids. The detailed behavior focused on the isotropic liquid phase is presented in Fig. 3. The negligible impact of nanoparticles on the clearing temperature is notable, although a set of reports shows an apparent shift [14, 15]. However, the shift appears in nanocomposites with the third component—a molecular surface agent connected to the nanoparticles to avoid sedimentation. Consequently, several ‘free’ molecular dopants must also exist in a three-component system. Notable that there is explicit experimental evidence that a molecular dopant in LC systems strongly changes the clearing temperature, also yielding the two-phase region between the isotropic liquid and LC mesophase [45, 46]. In this study, a surface agent is absent due to the focus on small ‘colloidal’ concentrations of NPs. Notable that a similar lack of a $T^{C}$ shift in nanocolloids composed solely of an LC compound and nanoparticles was reported earlier [23, 24, 31, 47, 48]. The lack of the third macromolecular component also prevented the introduction of disturbances into the BDS spectrum.

However, the significant influence of nanoparticles on the SmA–solid crystal transition appears. It can mean that the disordering introduced by the nanoparticles can be substantial: it takes place in the structured SmA phase but it is absent in the disordered Isotropic Liquid. Notable that despite the paraelectric nature of added BaTiO_3_ nanoparticles, they have a strong impact on the total value of the dielectric constant, mainly via the constant factor shift related to $\varepsilon^{*}$ in Eq. (1), as it is explicitly shown below. This shift is significant even for a tiny concentration of NPs, $x = 0.05\%$. For the isotropic liquid and solid crystal phases, the addition of 0.5% of nanoparticles led to an increase in the dielectric constant value in comparison to the pure 11CB (about 17% in the isotropic phase), but when reaching $x = 1\%$ concentration, the substantial drop of $\varepsilon$ is visible.

In the SmA phase, the pattern is different. The addition of nanoparticles first decreases the dielectric constant by ∼ 20% (for nanoparticles concentration $x = 0.05\%$), but for $x = 1\%$ concentration dielectric constant value increases up to ∼ 27% above that of pure 11CB.

**Fig. 4:**
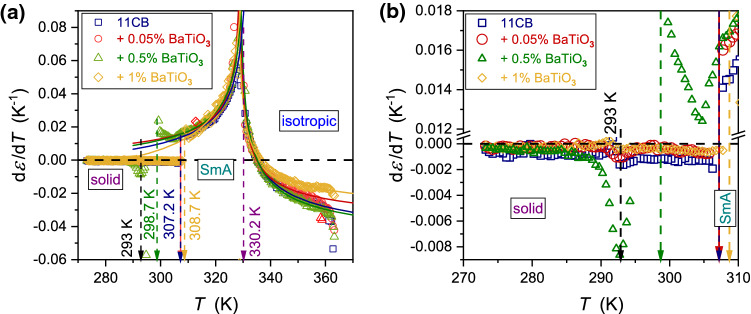
The derivatives of experimental data collected in Fig. 2 (derivatives of the temperature dependencies of dielectric constant in 11CB and related BaTiO_3_-based nanocolloids). The behavior in all tested phases is shown in Fig. 4a, and the focus on ${\text{Solid}} - {\text{SmA}}$ transition is presented in Fig. 4b

Figure 4 presents the derivative of experimental data given in Fig. 2. The plot reveals the range of pretransitional effects associated with $N \leftarrow \to I$, and ${\text{SmA}} \leftarrow \to N$ phase transitions: these effects cover the whole tested range of the isotropic liquid and smectic A phases, in both pure 11CB and nanocolloids. The nematic gap is too small (∼ 0.5 K) for a reliable test of pretransitional behavior, but it plays a role of an interesting ‘disturbation’ in the tests. The analysis of pretransitional effects was carried out in two steps. First, Eq. 2 was fitted to ${\text{d}}\varepsilon \left( T \right)/{\text{d}}T$ dependency in Fig. 4. Second, Eq. 1 was fitted to $\varepsilon \left( T \right)$ dependency in Fig. 2. In the second step, parameters obtained in the first step were used. Such an approach significantly increased the reliability of the nonlinear, multi-parameter fitting. Its results are shown as solid curves in Figs. 2, 3 and 4. Fitted parameters are collected in Table 1 for $N \leftarrow I$ transition and for ${\text{SmA}} \to N$ transition in Table 2. For $N \leftarrow I$ transition, the strong influence of NPs on the phase transition discontinuity $\Delta T^{*}$ is visible. For all tested systems, in the isotropic phase, the fit was optimal for the exponent value $\alpha = 0.5$. It is the hallmark feature of two ‘classic’ approaches: mean-field and tricritical-point (TCP) related. The latter is experimentally supported by the results of dielectric constant studies focused on the order parameter in 5CB and 8OCB [21, 29, 49], also containing the derivative analysis. They yielded the TCP order parameter exponent value $\beta = 1/4$ [21, 49]. Notable that for the systems described by mean-field model $\beta = 1/2$. The value of the exponent $\alpha = 0.5$ for the isotropic liquid phase is well established experimentally by dielectric constant and heat-capacity-related studies [5–10, 18–24, 29–31].

**Table 1:** Results of fitting Eqs. 1 and 2 to temperature dependencies of dielectric constant in the isotropic phase of 11CB and its nanocolloids with BaTiO_3_

	11CB	+ 0.05 (wt%)	+ 0.5 (wt%)	+ 1 (wt%)
ε^*^	12.2	11.1	13.0	9.7
*T*^C^(K)	330.2	329.7	330.2	330.7
T^*^(K)	327.3	328.4	328.3	327.9
ΔT(K)	3.0	1.3	2.0	2.9
*a*	− 0.055	− 0.043	− 0.056	− 0.036
*A*	0.31	0.22	0.29	0.21
α (fixed)	0.5	0.5	0.5	0.5

These results are shown graphically in Figs. 3 and 4

**Table 2:** Parameters obtained for $N - {\text{SmA}}$ pretransitional effect by fitting Eqs. (1) and (2) to experimental data

	11CB	+ 0.05 (wt%)	+ 0.50 (wt%)	+ 1 (wt%)
ε^*^	9.60	7.69	9.67	12.48
*T*^C^(K)	330.2	329.7	330.2	330.7
*T*^C^—0.5 = *T*_*N*−SmA_(K)	329.7	329.2	329.7	330.2
T^*^(K)	332.3	329.5	331.1	332.0
ΔT	2.6	0.3	1.4	1.8
*a*	0.017	0.006_4_	0.013	0.030
*A*	− 0.31	− 0.21	− 0.28	− 0.42
α (fixed)	0.5	0.5	0.5	0.5

These results are shown graphically in Figs. 2 and 4

The evidence for ${\text{SmA}} \to N$ transition is more complex. De Gennes, in his classic monograph [11], indicated the parallel between phase transitions in superconductors and $N - {\text{SmA}}$ transition in LC materials. For the *Physics of Critical Phenomena,* it means that both systems, different at the microscopic level, belong to the same universality class XY(2D) Heisenberg [4]. It is related to the specific heat critical exponent $\alpha \approx - 0.007$. However, experimental results of heat capacity measurements for $N - {\text{SmA}}$ transition are puzzling. The obtained values of the exponent range from $\alpha \approx - 0.007$ to $\alpha \approx 0.5$ [4, 50–52]. It is explained via the impact of the Fisher renormalization [53, 54], associated with the presence of an additional component, or by the empirical correlation to the ‘temperature width’ of the nematic phase, located between the isotropic liquid and the SmA phase [50]. In the given report, the value $\alpha \approx 0.5$ and the slightly discontinuous character of the $SmA \to N$ transition offer a fair portrayal of experimental data (Table 2) in all tested samples. However, nanoparticles notably influence the discontinuity $\Delta T^{*}$. The authors would like to draw attention to the recent evidence showing a split of the nematic phase in 8OCB into two domains ($N_{I} ,N_{{{\text{SmA}}}}$), associated with neighboring $I - N$ and $N - {\text{SmA}}$ transitions, respectively [31]. It is worth pointing out that the ‘width’ of the nematic phase in 8OCB: $\Delta T_{N} \approx 12\,{\text{K}}$ and the obtained exponent $\alpha = 0.11 - 0.2$ [31]. For 11CB the nematic phase width is tiny: $\Delta T_{N} \approx 0.5\,{\text{K}}$. Consequently, one can consider rather a $N_{I} - {\text{SmA}}$ phase transition for 11CB, and $N_{{{\text{SmA}}}} - {\text{SmA}}$ for 8OCB. It can offer additional comment regarding the origins of critical exponent values for $N - {\text{SmA}}$ transition in different systems.

Figure 4b focuses on the solid phase, which has been hardly discussed so far. Generally, for the melting/freezing transition, no pretransitional effects are expected [55]. Such behavior is also evidenced for LC-based materials discussed in the given report, except $x = 0.5\%$ nanocolloid, where a notable pretransitional effect appears in SmA (fluid) mesophase. Moreover, for this nanocolloid, the additional solid–solid transition also emerges. In the authors' opinion, the solid phase is probably associated with plastic crystalline phases. Such a statement can be supported by the mean value of the dielectric constant $\varepsilon \approx 4.5$ in this domain, as for a solid crystal with frozen translational and orientational arrangements $2 < \varepsilon < 2.5$ is expected.

Premelting effects on the solid side of the melting temperature $T_{m}$ are relatively often observed [55, 56]. They are not linked to the pretransitional phenomena but to the quasi-liquid channels forming near $T_{m}$, between solid grains [57]. The distortions-sensitive analysis in Fig. 4b reveals such behavior in systems discussed in this report and the possibility of strengthening this behavior by the added nanoparticles.

**Fig. 5:**
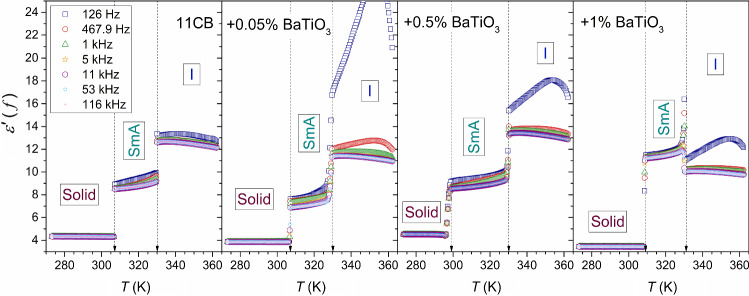
The real part of dielectric permittivity for a set of frequencies in the low-frequency domain in 11CB and its nanocolloids. The frequency range spans from the static domain, related to $f = 116\,{\text{kHz}}$, to $f = 126\,{\text{Hz}}$ in the LF domain

Figure 5 presents the temperature dependencies of the real part of dielectric permittivity for a set of frequencies, from the static to the low-frequency (LF) domain. Nanoparticles strongly influence $\varepsilon^{\prime } \left( f \right)$ value, with a decrease in the monitoring frequency. It is particularly visible in the isotropic liquid phase. This increase in $\varepsilon^{\prime } \left( f \right)$ value is associated with the apparent disappearance of the above-mentioned pretransitional effect.

**Fig. 6:**
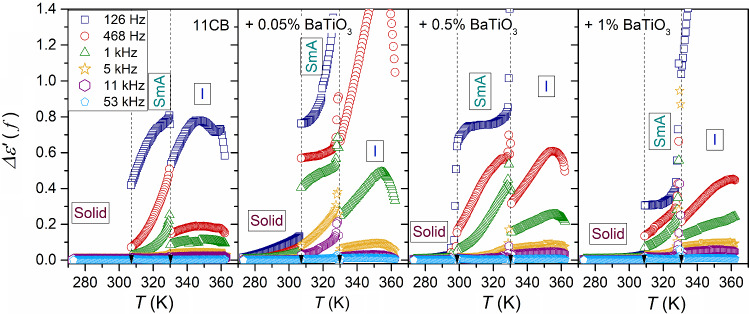
Temperature changes of the residual-ions contribution to the real part of dielectric permittivity emerging on decreasing frequency in the LF region, below the static domain; $\Delta \varepsilon^{\prime } \left( f \right) = \varepsilon^{\prime } \left( f \right) - \varepsilon$, in all tested phases of 11CB and its nanocolloids

**Fig. 7:**
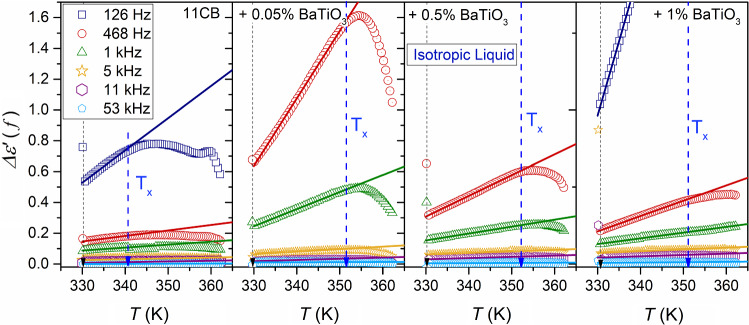
The focus on the isotropic liquid phase of 11CB and its nanocolloids: temperature changes of the residual-ions contribution to the real part of dielectric permittivity emerging on decreasing frequency in the LF region, below the static domain; $\Delta \varepsilon^{\prime } \left( f \right) = \varepsilon^{\prime } \left( f \right) - \varepsilon$

**Fig. 8:**
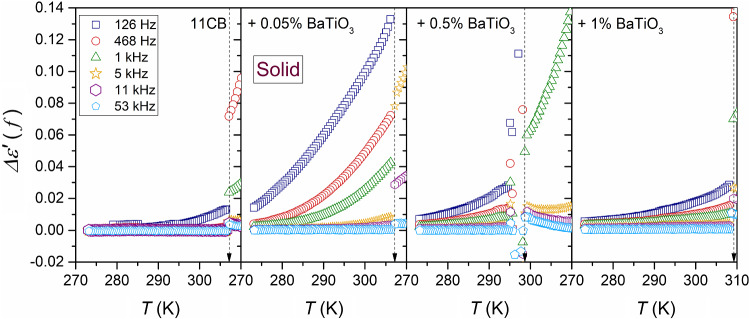
The focus on the solid phase of 11CB and its nanocolloids: temperature changes of the residual-ions contribution to the real part of dielectric permittivity emerging on decreasing frequency in the LF region, below the static domain; $\Delta \varepsilon^{\prime } \left( f \right) = \varepsilon^{\prime } \left( f \right) - \varepsilon$

Notwithstanding, the impact of pretransitional fluctuations becomes explicitly visible if data from Fig. 5 are considered in frames of the ‘difference analysis,’ introduced by Eq. 6. This analysis method yields insight that is limited to the ionic-related contribution. Such results are presented in Fig. 6 for all tested phases of 11CB and its nanocolloids. Figures 7 and 8 show the behavior focused on the isotropic liquid and solid phases, respectively. For the isotropic phase, the linear behavior, suggested by Eqs. 5 and 6, is visible. Such behavior is strongly enhanced when adding nanoparticles. Even a tiny addition of NPs (0.05%) can significantly increase the ionic contribution to dielectric permittivity in comparison to pure 11CB, and this impact diminishes when increasing NPs concentration. The evidence for mentioned linear behavior was earlier obtained in the isotropic phase of 5CB, where it occurred up to $T_{x} \sim T^{C} + 40 {\text{K}}$ [41]. Such simple behavior emerging on approaching I–N transition in 5CB was explained by the influence of prenematic fluctuations. Notable that the value of $T_{x}$ correlates with the termination of detecting the precritical features by methods directly coupled to fluctuations, such as NDE for instance [43]. However, in 5CB the mesomorphism solid–nematic–isotropic takes place. In 11CB pretransitional fluctuations have to exhibit strong pre-smectic features, which can comment on the shortening of the mentioned linear behavior.

In the SmA phase, notable are (very) strong pretransitional changes of $\Delta \varepsilon^{\prime } \left( {f,T} \right)$ for ${\text{SmA}} \to N$ transition. The discussed way of analysis also leads to the emergence of pretransitional effects for ${\text{Solid}} \left( {{\text{crystal}}} \right) \leftarrow \left( {{\text{fluid}}} \right) {\text{SmA}}$ transition, as well as ${\text{Crystal}} \to {\text{SmA}}$. One should recall that $\Delta \varepsilon^{\prime } \left( {f,T} \right)$ is related solely to the ionic contribution of the electric polarizability, whereas permanent dipole moments contribute mainly to the dielectric constant.

When discussing dielectric properties in the low-frequency domain, one cannot omit DC electric conductivity, being a ‘dynamic equivalent’ of the dielectric constant, related to $\varepsilon^{\prime \prime } \left( f \right)$, as follows [16, 17]:

7 $$ \sigma_{{{\text{DC}}}} = \sigma = \varepsilon^{\prime \prime } \left( f \right) = 2\pi f\varepsilon^{\prime \prime } \left( f \right). $$

It means that a straight line with a slope of − 1 should appear on the log–log scale in the LF part of $\varepsilon^{\prime \prime } \left( f \right)$ spectrum, as in Fig. 1.

**Fig. 9:**
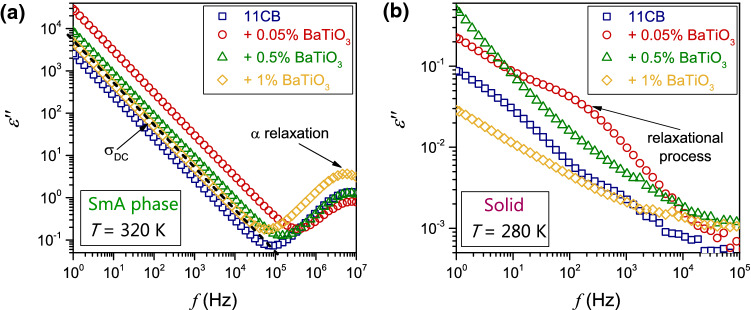
The imaginary part of dielectric permittivity spectra focused on the low-frequency domain for SmA phase **a** and the solid phase **b** in 11CB and its nanocolloids with BaTiO_3_

**Fig. 10:**
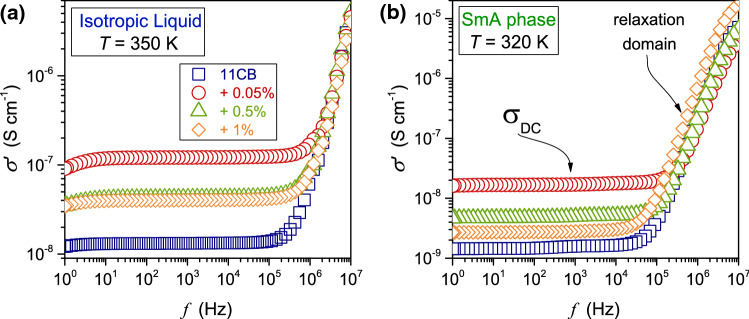
The real component of the complex electric conductivity obtained from data shown in Fig. 1 for the SmA phase and the isotropic liquid phase. Results were obtained via Eq. 9, for 11CB and its nanocolloids. The ‘stationary’ (frequency-independent) and relaxation domains are indicated

Figure 9a, b presents such behavior for the SmA mesophase and solid phase in 11CB. For the SmA phase, Eq. 7 is perfectly fulfilled, but for the solid phase, it is distorted by LF relaxational processes and the Maxwell–Wagner polarization effect. In such a case, Eq. 7 cannot be used for estimating DC electric conductivity. The ultimate test for the reliable determining DC electric conductivity constitutes the transformation for the complex dielectric permittivity representation, as shown in Fig. 10. The horizontal part in the plots, for SmA and Isotropic liquid phase is DC electric conductivity.

The primary relaxation time and DC electric conductivity are linked via the Debye–Stokes–Einstein (DSE) relation [58]:

8 $$ \tau \left( T \right)\sigma \left( T \right) = C \Rightarrow \frac{\tau \left( T \right)}{{C\sigma^{ - 1} \left( T \right)}} = 1 \Rightarrow \frac{\tau \left( T \right)}{{\tau_{\sigma } \left( T \right)}} = 1. $$

The right-side part of Eq. 8 is for the basic DSE equation. In complex systems, particularly near the glass transition, the exponent showing the translational–orientational (T&O) decoupling can appear, associated with the exponent S coupled to the orientational relaxation time. The right-side part of Eq. 8 shows that the reciprocal of DC electric conductivity is the metric of the translational ($\sigma$-related) relaxation time. Consequently, the following general evolution of discussed properties can be expected [16]:

9 $$ \sigma_{DC}^{ - 1} \left( T \right),{ }\tau \left( T \right),{ }\tau_{\sigma } \left( T \right) \propto {\text{exp}}\left( {\frac{{E_{a} \left( T \right)}}{{{\text{RT}}}}} \right) $$

where $E_{a} \left( T \right)$ is the apparent (temperature dependent) activation energy, and *R* stands for the gas constant.

Equation 9 might be simplified to the basic Arrhenius pattern if $E_{a} \left( T \right) = E_{a} = const$, in the given temperature domain. In such a case, one obtained the linear domain for the so-called Arrhenius plot [16]. For instance:

10 $$ {\text{log}}_{10} \sigma^{ - 1} \left( T \right) = \frac{{{\text{ln}}\sigma_{0}^{ - 1} }}{{{\text{ln}}10}} + \frac{{E_{a} }}{{{\text{RTln}}10}} = A + B \times \frac{1}{T} $$

where $\sigma_{0}^{ - 1}$ is the prefactor in Eq. 9; constant parameters: $A = \ln \sigma_{0}^{ - 1} /\ln 10$ and $B = E_{a} /R\ln 10$.

**Fig. 11:**
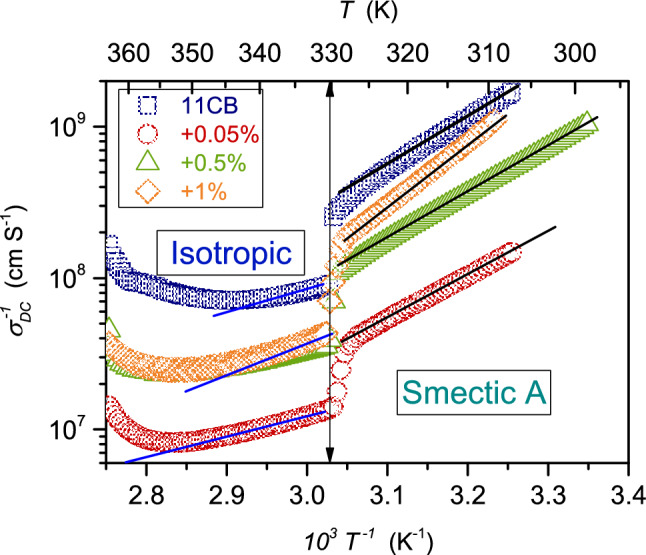
Reciprocals of DC electric conductivity in a function of reciprocal temperature in the isotropic and SmA phase of 11CB and its nanocolloids

If the T&O decoupling takes place, $E_{a}^{\alpha } \left( T \right) = {\text{SE}}_{a} \left( T \right)$ appears in Eq. 9 [59]. Figure 11 shows the Arrhenius plot for the reciprocal of DC electric conductivity in the isotropic liquid and SmA phase of 11CB and its BaTiO_3_-based nanocolloids. The linear behavior in the SmA phase indicates the basic Arrhenius pattern, with the constant activation energy and a minor pretransitional distortion near the N–SmA transition.

For the isotropic liquid, such behavior is visible only for $x = 0.05\%$ nanocolloid. For other tested nanocolloids and pure 11CB, the super-Arrhenius behavior with apparent (changeable) activation energy takes place. It can be linked to the joined impact of pretransitional, pre-LC fluctuations, and nanoparticles. Following Eqs. 9 and 10, one should expect the systematic rise of $\sigma_{{{\text{DC}}}}^{ - 1} \left( T \right)$, $\tau_{\sigma } \left( T \right)$, and $\tau \left( T \right)$ on cooling. Indeed, such behavior is evidenced in Fig. 11, except for the high-temperatures limit in the isotropic liquid. The authors do not have an explanation for this phenomenon.

Finally, we would like to address Eqs. (3) and (4), which are often recalled when discussing dielectric spectra in the LF domain [32–40]. The above discussion validated Eq. 4, showing its link to DC electric conductivity: note the direct relationship between Eqs. (4) and (7). Hence, Eq. (4) simply reflects the definition of DC electric conductivity. Its violation can be associated with the emergence of low-frequency relaxation processes or layer-polarization effects, such as the Maxwell–Wagner effects.

As for Eq. (3), the authors have not found its explicit experimental validation. Notwithstanding, it is suggested that experimental data obey Eq. 3 in a frequency range from a few Hz, or in some reports ∼ 100 Hz, to about 100 kHz. In Fig. 1, complex dielectric permittivity spectra in tested LC-based systems are presented. These spectra show that the static domain extends from a few kHz to more than 1 MHz. On decreasing frequency, until reaching ca. 100 Hz the impact of the ionic contribution remains weak, and the ions-related boost of $\varepsilon^{\prime } \left( f \right)$ occurs for $f < 100\,{\text{Hz}}$. Hence, the behavior of the real part of dielectric permittivity in the static domain (dielectric constant) can be considered a specific non-ionic background, as indeed results from Eq. 3. It enabled the focus solely on the ionic contribution and validation by the simple scaling plot:

11 $$ (\varepsilon^{\prime } \left( f \right) - \varepsilon )f^{3/2} = \Delta \varepsilon^{^{\prime}} \left( f \right)f^{3/2} = \left( {\frac{{nq^{2} D^{3/2} }}{{\pi^{3/2} \varepsilon_{0} Lk_{B} T}}} \right) = A = {\text{const}}{.} $$

**Fig. 12:**
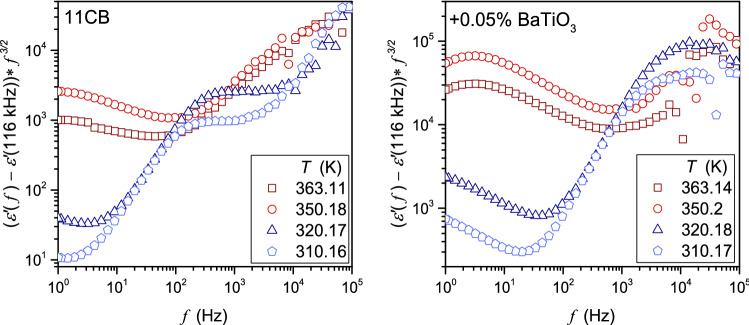
The test of Eq. 12, suggesting the universal frequency-related behavior of the real part of dielectric permittivity in the ionic-dominated LF domain (Eq. 3): results for 11CB and its nanocolloid in the isotropic liquid and SmA, phases. The lack of horizontal behavior clearly shows the lack of the universality suggested by Eq. 3

Figure 12 presents the results of such analysis. No horizontal line, suggested by Eq. (11) in reference reports appears. Hence, the test of Eq. 3 for the LF behavior of the real part of dielectric permittivity is negative. This conclusion covers the tested in Fig. 12 frequency range from 1 Hz to 100 kHz, as well as frequency sub-ranges. In the opinion of the authors, the re-analysis of Eq. (3) in frames of its theoretical background is required [35, 60]. The question also arises about the possible frequency dependence of the diffusion coefficient in Eqs. (3) and (11). Generally, diffusion is coupled to the electric conductivity, namely $D \propto \sigma$ or $D \propto \sigma /T$, as shown by Debye–Stokes–Einstein (DSE) relations [58]. Additionally, considering Eq. (7) defining DC conductivity, one obtains a supplementary contribution to the frequency dependence in Eqs. (3) and (11). In the presence of heterogeneities in the ‘uniform’ fluid surrounding, the fractional (fDSE) form may be expected, namely: $D \propto \left( \sigma \right)^{\xi }$ or $D \propto \left( {\sigma /T} \right)^{\xi }$. , where $\xi$ is the fractional DSE exponent [7, 31, 58]. The role of heterogeneities may be played by pretransitional fluctuations and also nanoparticles. It can open the possibility of testing their impact on spectra-related frequency dependences.

## Conclusions

This report shows the analysis of the temperature behavior of the real part of dielectric permittivity in the static (dielectric constant) and low-frequency domains in bulk samples of 11CB and its BaTiO_3_-based nanocolloids, extending from the isotropic liquid, through smectic A to the solid phase. In each phase, the dominant impact of pretransitional fluctuations was detected, significantly moderated by nanoparticles. The authors propose to split focus on the dielectric constant ${\upvarepsilon }\left( {\varvec{T}} \right)$ evolution, yielding mainly response from permanent dipole moment and its arrangement, and in the LF domain $\Delta \varepsilon^{\prime } \left( f \right) = \varepsilon^{\prime } \left( f \right) - \varepsilon$ which is associated solely with ionic-related polarization mechanisms. The distortions-sensitive analysis is also worth mentioning, as it offers insight into local distortion within the tested temperature dependence. All of these led to new experimental evidence for I–N, N–SmA, and SmA–solid transitions in tested systems. This evidence includes the extent of pretransitional effects (i.e., fluctuations-dominated domains), critical exponent, and phase transitions discontinuities. Notable is the evidence of pretransitional effects for the SmA–solid crystal transition, particularly for $\Delta \varepsilon^{\prime } \left( {{\varvec{f}},{\varvec{T}}} \right)$ evolutions. In the authors' opinion, this issue is worth stressing since the transition from the LC mesophase to the solid phase still constitutes a cognitive gap. Finally, we would like to highlight the validating discussion of Eqs. (3) and (4), which are often recalled as the reference in discussing complex dielectric permittivity in the low-frequency domain.

## Data Availability

The datasets generated during and/or analyzed during the current study are available from the corresponding author on reasonable request.
